# Image-Based Bolt-Loosening Detection Using a Checkerboard Perspective Correction Method

**DOI:** 10.3390/s24113271

**Published:** 2024-05-21

**Authors:** Chengqian Xie, Jun Luo, Kaili Li, Zhitao Yan, Feng Li, Xiaogang Jia, Yuanlai Wang

**Affiliations:** 1 School of Civil Engineering and Architecture, Chongqing University of Science and Technology, No. 20, East University Town Road, Shapingba District, Chongqing 401331, China; 2021206118@cqust.edu.cn (C.X.);; 2 Chongqing Urban Investment Infrastructure Construction Co., Ltd., Chongqing 400015, China

**Keywords:** flange joints, bolt loosening, perspective correction, checkerboard, camera calibration

## Abstract

In this paper, a new image-correction method for flange joint bolts is proposed. A checkerboard is arranged on the side of a flange node bolt, and the homography matrix can be estimated using more than four feature points, which include the checkerboard corner points. Then, the perspective distortion of the captured image and the deviation of the camera position angle are corrected using the estimated homography matrix. Due to the use of more feature points, the stability of homography matrix identification is effectively improved. Simultaneously, the influence of the number of feature points, camera lens distance, and light intensities are analyzed. Finally, based on a bolt image taken using an iPhone 12, the prototype structure of the flange joint in the laboratory is verified. The results show that the proposed method can effectively correct image distortion and camera position angle deviation. The use of more than four correction points not only effectively improves the stability of bolt image correction but also improves the stability and accuracy of bolt-loosening detection. The analysis of influencing factors shows that the proposed method is still effective when the number of checkerboard correction points is reduced to nine, and the average error of the bolt-loosening detection result is less than 1.5 degrees. Moreover, the recommended camera shooting distance range is 20 cm to 60 cm, and the method exhibits low sensitivity to light intensity.

## 1. Introduction

Bolt loosening in bolted joints can affect overall structural safety. Previously, bolt-loosening detection was primarily manual, conducted by maintenance personnel by observing the appearance of the bolt, utilizing a hammer to strike the bolt, and measuring the bolt torque using a torque wrench [1]. However, manual detection is inefficient, cannot achieve real-time detection, and poses a significant danger in large-scale engineering structures.

Various scholars have advocated using online sensors to address the shortcomings of manual bolt-loosening detection. The principal techniques include guided wave, utilization of piezoelectric sensors, impedance technology, and computer vision technology. The methods of guided wave are based on the wave theory, using the propagation characteristics of waves in materials to detect the loosening of bolts as well as stress changes in the bolt structure [2,3,4]. The piezoelectric-sensor-based method detects bolt loosening by monitoring variations in the ultrasonic transmission energy loss at the interface of connected elements. Its use is widely recognized due to the sensor’s compact form, ease of installation, and capability to identify online system faults effectively [5,6,7]. Bolt-loosening detection employing impedance technology hinges on the piezoelectric qualities of piezoelectric materials, which exhibit altered impedance characteristics, as bolt looseness translates to reductions in structural stiffness and increases in damping [8,9,10,11,12]. The key drawback of this method is its costliness and the necessity for an extensive array of sensors. Conversely, a computer vision-based approach requires only a high-definition camera to capture images of the bolts, thus facilitating loosening detection. Coupling this method with deep learning drastically enhances the efficiency and accuracy of image target recognition. The integration of deep learning further boosts the method’s detection effectiveness and stability. Consequently, a vision-based technique for bolt-loosening detection holds great prospects for advancement.

**Figure 1:**
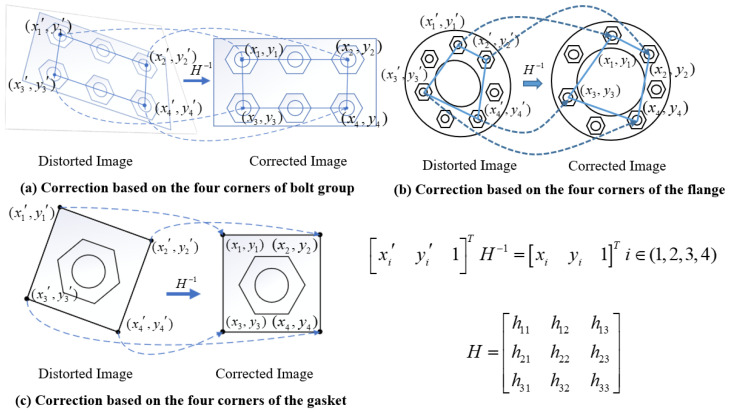
Schematic diagrams of bolt image-correction methods.

Currently, computer vision technology has been widely used in the fields of construction, engineering management, structural damage identification, and operation and maintenance. This includes, for example, building construction and management applications based on computer vision technology [13,14,15]. Others include those based on image processing technology to identify structural crack detection and safety assessments of concrete structures, road bridges, and steel structures [16,17,18]. Bolt-loosening detection methods based on computer vision technology are classified into three types: image processing technology, deep learning, and both deep learning and image processing technology. For example, based on image processing technology, the four corner points of a bolted connection plate have been used to correct the perspective distortion of the bolt image, and the Hough transform and Canny edge detection operator have been utilized to identify the nut edge line to calculate the rotation angle [19,20]. Bolt looseness has been diagnosed based on deep learning. For example, Zhang et al. [21] proposed a method to identify changes in screw height based on deep learning. Wang et al. [22] and Pal et al. [23] proposed a method for identifying vibration time–frequency spectra of bolt structures based on deep learning. Wang et al. [24] detected the corrosion and loosening of bolts based on an improved YOLOv5s target detection model, mainly enhancing the feature extraction module and introducing the linear detector VIT. Bolt looseness has been diagnosed using deep learning and image processing technology. For instance, Huynh et al. [25] proposed identifying and segmenting bolt images based on a CNN, correcting distortion based on the perspective image of the bolt coordinates of the four corner points on the bolt connection, and calculating the loosening angle based on the bolt edge line. Coelho et al. [26] proposed a combined unsupervised and supervised machine-learning algorithm architecture for pattern recognition, detection, and quantification of torque loosening in bolted connections. Wang et al. [27] proposed that bolt images be perspective-corrected using the four bolt center points in the flange connection bolt images. Luo J et al. [28] proposed that the structure of the flange joint is unique, necessitating the use of a single bolt as the object of inspection. Furthermore, the bolt image is corrected based on the four corner points of the rectangular washer of the individual bolt. However, the abovementioned methods still need to be improved as they have many limitations. For example, image-correction problems are influenced by camera position. The goal of image correction is to correct an image’s rotation and perspective distortion angles caused by camera position and shooting angle. Image correction is a key step in detecting engineering problems based on image processing technology, and it is related to the reliability of the inspection results. The proposed method for the perspective correction of bolt images is divided into two types according to the bolt structure. One type is to correct perspective distortion based on the four bolt points of the bolt group when the detection object is the bolt group [25,29], as shown in Figure 1a. The second type is to correct perspective distortion based on the four bolt points on the flange or the four corner points of the rectangular gasket of a single bolt when the detection object is a flange node [27,28], as shown in Figure 1a,c. These methods are used to calculate the homography matrix for perspective distortion correction based on the extraction of four coordinate points in the image.

**Figure 2:**
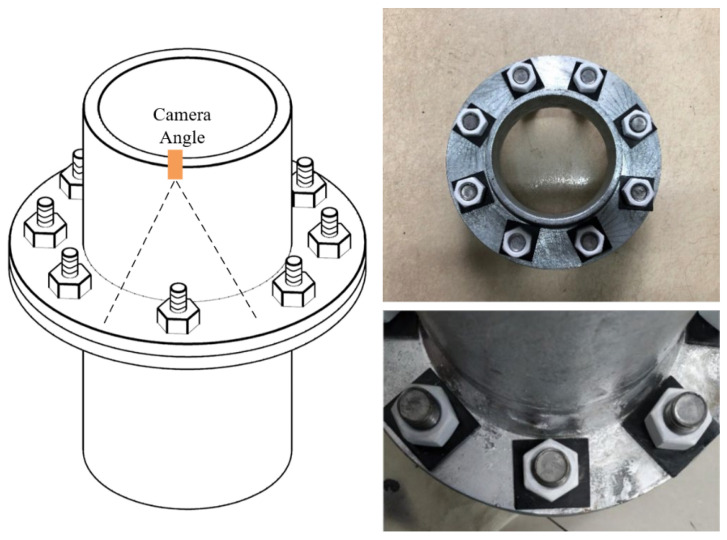
Flange joints and bolt images.

However, due to the special structure of the flange joint and the occlusion of the connecting rods in the structure, the two methods presented in Figure 1a,b are unsuitable for correcting flange bolt images. Only the method shown in Figure 1c is applicable. There are, however, several issues with the correction method illustrated in Figure 1c. Firstly, the deviation in the camera position cannot be corrected. Secondly, the extraction of the four corner points of the gasket fails when shadow occlusion is present [30]. Lastly, it is difficult to achieve rectangularization of the gaskets in the existing flange connection nodes, and the existing distortion correction methods cannot be directly applied to the flange connection nodes that are already in place. A rectification schematic diagram of these methods is shown in Figure 1. A flange joint diagram is shown in Figure 2.

In this paper, the influence of camera position deviation on flange node bolt image correction is summarized, and a new flange node bolt image-correction method is proposed, which can effectively correct image perspective distortion and camera position deviation. This method is based on the inner corner points of the checkerboard and combined with the homography matrix distortion correction algorithm to construct a bolt image distortion correction algorithm suitable for existing flange connection nodes. First, the camera’s internal parameters are corrected to rectify lens distortion. Then, the homography matrix of multiple correction points is calculated based on the corner points of the checkerboard to correct the image.

The sections of this paper are arranged as follows: The Section 2 introduces the limitations of rectangular spacers in correcting camera position deviations. The Section 3 introduces the checkerboard-based image-correction method proposed in this article. The Section 4 verifies the feasibility and stability of image perspective correction using the proposed method through experiments. It analyzes the influence of different numbers of correction points, camera shooting heights, and light intensities. The Section 5 summarizes the article.

## 2. Limitations of Rectangular Spacer Method for Correcting Camera Position Deviation

**Figure 3:**
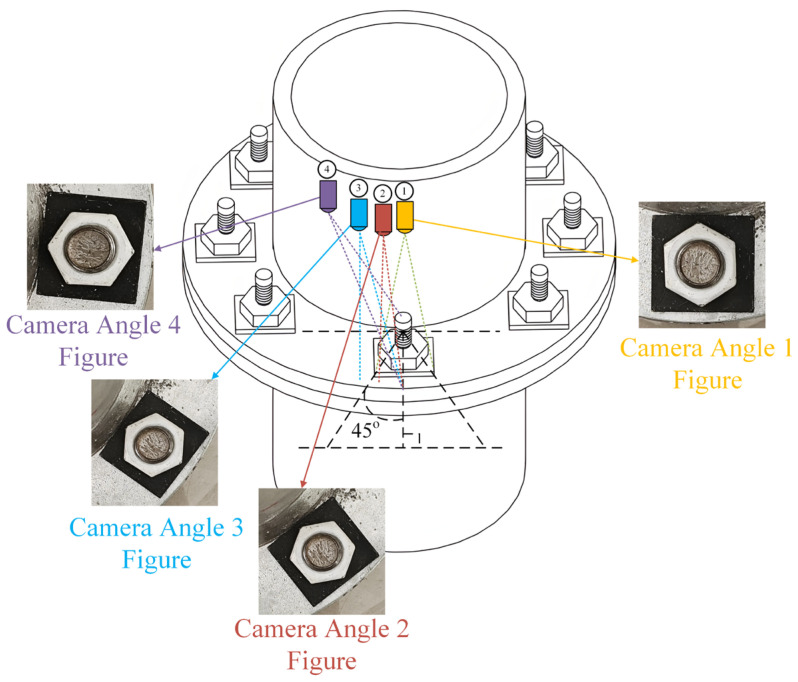
Images taken at different camera positions.

In image-based bolt-loosening detection methods, using dynamic camera collection methods, such as drones, can cause image perspective distortion and affect image rotation angles due to deviations in camera shooting angles and positions. Image perspective distortion distorts the bolt edges in an image, and positional deviation directly affects the rotation angle of the bolt in the image. Both factors can impact the accuracy of the test results and, thus, necessitate correction. Currently, detection methods based on the four corner points of rectangular gaskets can correct the image’s perspective distortion but cannot effectively correct the influence of deviations in camera position. Bolt images captured using four camera positions, simulating real-world image collection, are shown in Figure 3. The deviation angles of camera positions 1, 2, 3, and 4 are close to 0 degrees, slightly less than 45 degrees, slightly greater than 45 degrees, and slightly less than 90 degrees, respectively.

**Figure 4:**
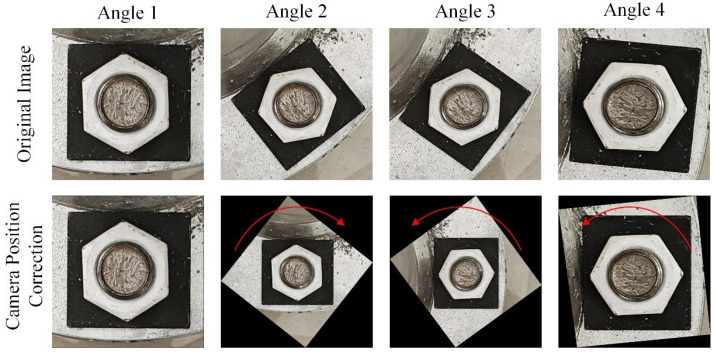
Image results captured at different camera positions corrected based on four corner points of rectangular spacer.

Figure 4 shows the result of using the four corner points of a rectangular gasket to correct the bolt image taken in Figure 3. Figure 4 illustrates that the image-correction method based on the four corner points of the rectangular gasket can effectively correct deviations in the position of the image when the deviation angle of the camera position is less than 45 degrees.

However, when the deviation angle of the position is greater than 45 degrees, the image is corrected based on the rectangular spacer, but the corrected image is significantly different from the initial image, with a phase of 0 degrees, and there is a 60-degree rotation between the two images. The difference in angle may negatively impact diagnosing bolt looseness. Therefore, the correction results of Angles 3 and 4 in Figure 4 have an error of nearly 60 degrees, while the results of Angles 1 and 2 are very close to the true bolt angle.

In conclusion, the rectangular-spacer-based correction method is unable to rectify the errors stemming from the camera position. Since camera positioning significantly influences the accuracy of detection results, it is essential to address and correct any deviations.

## 3. Checkerboard-Based Bolt Image-Correction Method and Bolt-Loosening Diagnosis

### 3.1. Checkerboard-Based Bolt Image-Correction Method

The proposed checkerboard-based bolt image-correction method can effectively solve the influence of deviations in camera position, which cannot be solved via all current methods. The main principle is that checkerboards have more correction points than rectangular shims. Therefore, a checkerboard can provide more correction pixel coordinates when correcting an image. Based on these pixel coordinates, the ideal position state of the checkerboard pixel position can be established; then, the image is corrected through homography transformation. Additionally, the number of correction points can be specified arbitrarily, the correction direction required for the image can be easily determined based on the shape and layout of the correction points, and the image can always be corrected in the proposed positive direction.

**Figure 5:**
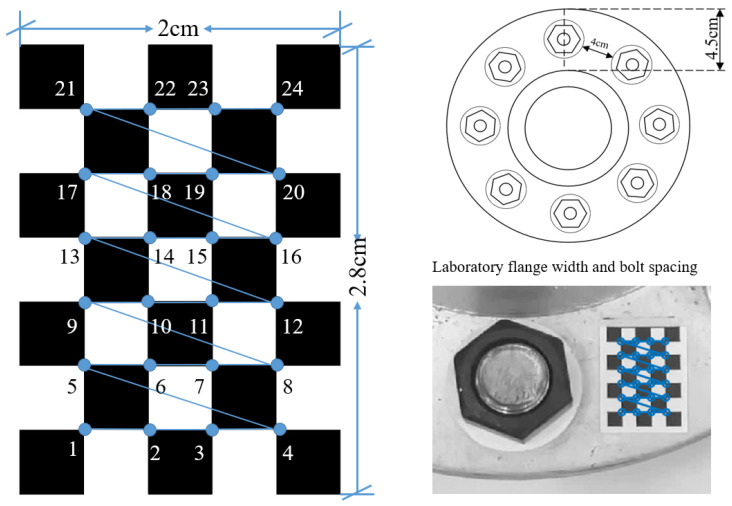
Checkerboard and device sizes.

A diagram of the correction device is shown in Figure 5. The device is used to paste a checkerboard with a size of 2 cm × 2.8 cm between the flange joint bolts and the bolts. The checkerboard has five columns and seven rows, and the size of each rectangle is 0.4 mm. The size of the checkerboard can be adjusted according to the size of the flange and the distance between the bolts. This method aims to obtain camera parameters through checkerboard camera calibration to correct internal camera parameters. The corner points of the checkerboard are identified based on the corrected bolt image, and the homography matrix is calculated based on the checkerboard’s corner points to correct the perspective distortion of the bolt image and deviations in camera position. The proposed method can use more than four coordinate points to calculate the homography matrix. Compared with the existing methods, the stability and accuracy of the correction are effectively improved.

**Figure 6:**
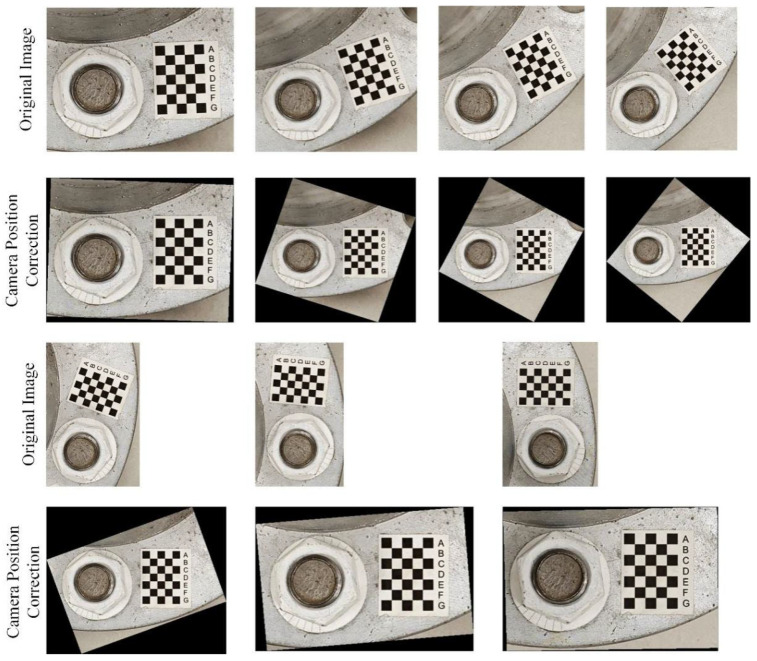
Correcting deviations in the bolt image position based on the checkerboard method.

Using the proposed checkerboard image-correction method, images with deviations in position from 0 to 90 degrees on one side were corrected, and the results are shown in Figure 6. These results show that all images have been effectively corrected, so it can be concluded that the proposed correction method can correct deviations in position from 0 degrees to 180 degrees.

### 3.2. Checkerboard-Based Bolt Image-Correction Method Process

**Figure 7:**
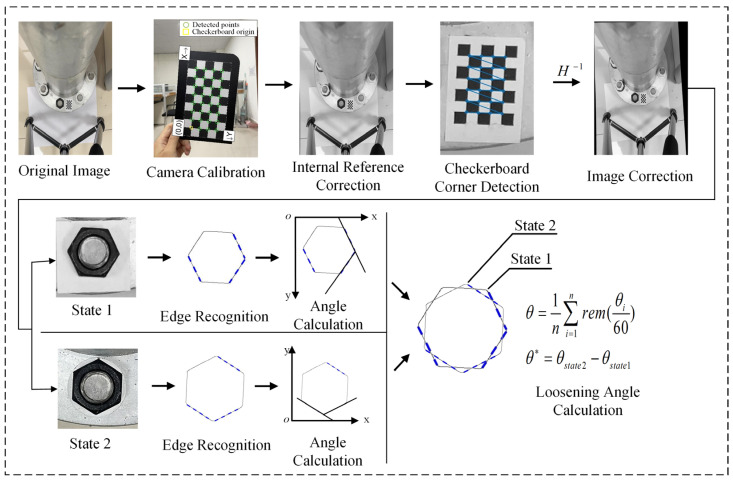
Method and program diagram.

A program flowchart of the proposed method is shown in Figure 7. Firstly, the internal parameters of the camera are used to correct the lens distortion of the original image. These parameters are obtained via camera calibration [31]. Then, perspective distortion correction is performed based on the corrected internal reference image. The program can automatically detect the checkerboard in the input image and identify the corner coordinates of the checkerboard. The homography [32,33] matrix is calculated based on the corner coordinates of the checkerboard to correct distortion in the bolt image. The homography matrix is a mapping transformation matrix with a size of 3 × 3. A minimum of four coordinate points is required to map one plane to another. However, due to the influence of test errors and pixel precision, there are certain errors in the extraction of the four coordinate points. Therefore, only four points are used for image correction, so there may be a large correction deviation. The proposed method uses more landmarks for distortion correction. Theoretically, the more correction points used, the smaller the correction error and the better the stability. The formula for calculating the homography matrix of more than four correction points is as follows:

(1) $${\left[\begin{array}{cccccccc} {x_{1}}^{\prime } & {y_{1}}^{\prime } & 1 & 0 & 0 & 0 & -x{x_{1}}^{\prime } & -x{y_{1}}^{\prime }\\ 0 & 0 & 0 & {x_{1}}^{\prime } & {y_{1}}^{\prime } & 1 & -{x_{1}}^{\prime }y & -y{y_{1}}^{\prime }\\ & & & & \cdot & & & \\ & & & & \cdot & & & \\ & & & & \cdot & & & \\ & & & & \cdot & & & \\ {x_{n}}^{\prime } & {y_{n}}^{\prime } & 1 & 0 & 0 & 0 & -x_{n}{x_{n}}^{\prime } & -x_{n}{y_{n}}^{\prime }\\ 0 & 0 & 0 & {x_{n}}^{\prime } & {y_{n}}^{\prime } & 1 & -x_{n}{y_{n}}^{\prime } & -y_{n}{y_{n}}^{\prime } \end{array}\right]}_{2n\times 8}{\left[\begin{array}{c} h_{11}\\ h_{12}\\ h_{13}\\ h_{21}\\ h_{22}\\ h_{23}\\ h_{31}\\ h_{32} \end{array}\right]}_{8\times 1}={\left[\begin{array}{c} x\\ y\\ •\\ •\\ •\\ •\\ x_{n}\\ y_{n} \end{array}\right]}_{2n\times 1}$$

where (*x*, *y*) are the world coordinates of the correction point, and (*x*′, *y*′) are the image coordinates corresponding to the correction point. It can be seen that when the number of correction points is greater, the result of the calculated homography matrix is more accurate. Finally, after perspective correction, the nut edge in the two states of the bolt image is identified based on the Canny edge detection operator and Hough transform. The bolt rotation angle is calculated using the established damage index [30].

## 4. Method Test Verification

### 4.1. Test Verification

It has been verified that the proposed checkerboard correction method can correct the impact of deviations in camera position. In order to verify the effectiveness and stability of the proposed method in correcting image perspective distortion, a bolt image was corrected based on a laboratory checkerboard, and the loosening angle was calculated for the corrected bolt image. Finally, the validity and stability of the method were obtained by comparing the loosening angles calculated from the corrected and uncorrected bolt images.

**Figure 8:**
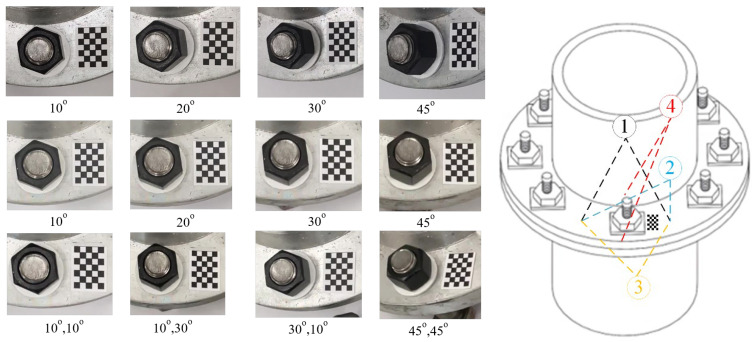
Bolt images under different perspective angles.

**Figure 9:**
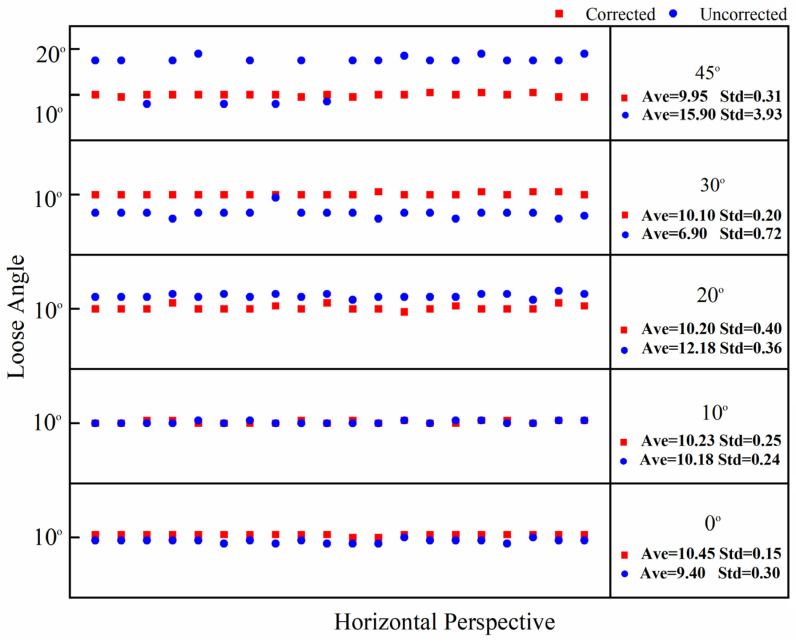
Calculation data map of 20 horizontal perspective bolt images with corrected and uncorrected bolt looseness values.

**Figure 10:**
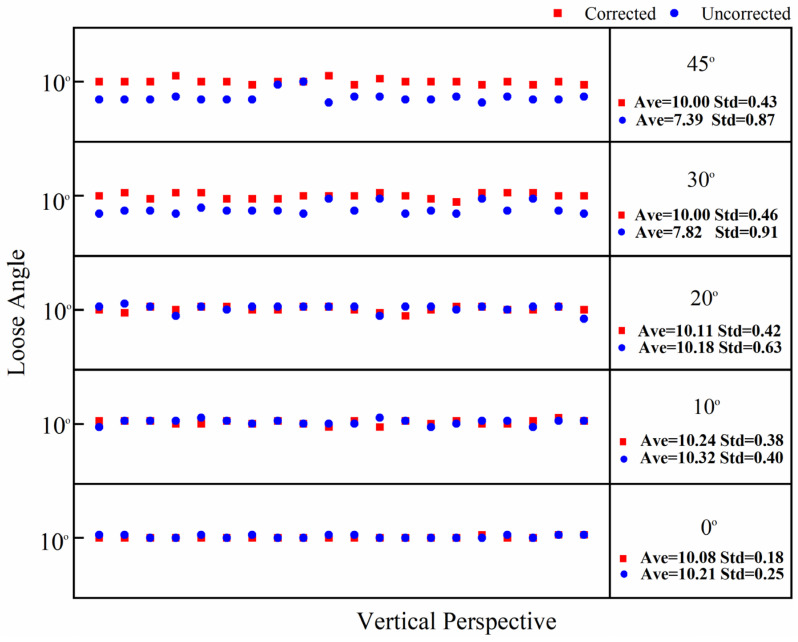
Calculation data map of 20 vertical perspective bolt images with corrected and uncorrected bolt looseness values.

**Figure 11:**
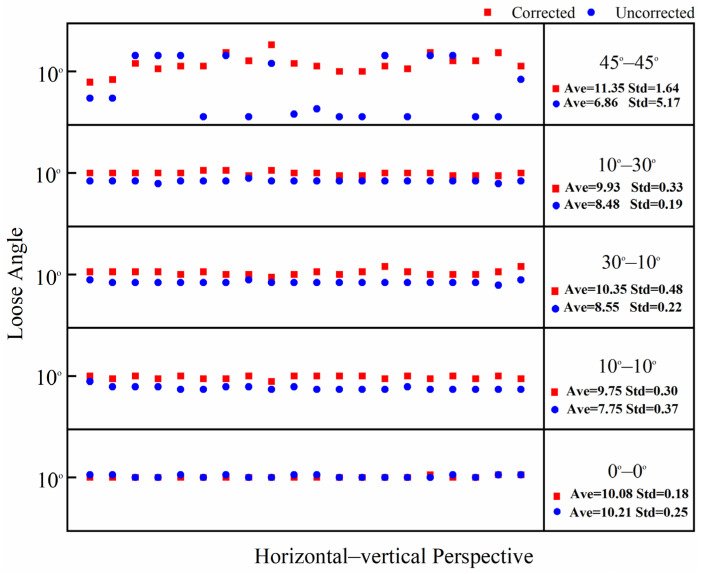
Calculation data map of 20 horizontal–vertical perspective bolt images with corrected and uncorrected bolt looseness values.

In this test, the bolt damage was set to 10°. The experiment utilized the following conditions: without a perspective angle, with a horizontal perspective angle, with a vertical perspective angle, and with a horizontal–vertical perspective angle. The group without a perspective angle was the control group. The horizontal and vertical perspective angles were changed from 10° to 20°, 30°, and 45°. The horizontal–vertical perspective angles were changed from 10°–10° to 10°–30°, 30°–10°, and 45°–45°. The camera shooting height was 55 cm. The checkerboard used in the experiment is shown in Figure 5. The bolt images under different perspective angles are shown in Figure 8. In order to ensure that the checkerboard was photographed, we utilized four viewing angles, i.e., viewing angles 1, 2, 3, and 4. Viewing angle 1 is a non-perspective angle, and angle 2 is a two-way perspective shooting angle. Viewing angle 3 is a vertical perspective shooting angle. Viewing angle 4 is a horizontal perspective shooting angle. The side length of a single checkerboard rectangle was 0.4 cm, with 7 rows and 5 columns, an area of 5.6 cm^2^, and 24 correction points. The test images were taken using an iPhone 12. Twenty images were tested for each set of perspective angles. The specific test conditions are shown in Table 1. The test results are shown in Figure 9, Figure 10 and Figure 11. Ave represents the mean value of 20 test results. Std is the standard deviation of the 20 test results.

The test results show that when the horizontal perspective angle is greater than or equal to 20°, the vertical perspective angle is greater than or equal to 30°, and when the horizontal–vertical perspective angle is greater than 10°, the accuracy and stability of the corrected bolt image are significantly improved compared with the uncorrected bolt image. The overall average error of the loosening angle calculated from the corrected bolt image is less than 1.5°, and the data stability is excellent. The overall average error of the loosening angle calculated from the uncorrected bolt image is 5.9° at most, and the error and stability of loose angle detection increase with the perspective angle. Thus, through comparison, it can be clearly concluded that the proposed bolt image-correction method based on the checkerboard exhibits a good correction effect as well as stability and can improve the stability and diagnostic accuracy of bolt-loosening diagnosis.

### 4.2. Experimental Comparison of Checkerboard-Based and Rectangular-Spacer-Based Bolt Image-Correction Methods

In order to verify the advantages of the proposed checkerboard-based correction method compared to the existing rectangular-spacer-based correction method, tests were conducted on both methods under the same experimental conditions. A 10° loosening angle was considered in the experiment, and 20 images were taken under the 10° looseness condition. One image was taken under the initial (unfastened) condition, which can be used as the initial image for calculating the bolt-loosening angle.

**Figure 12:**
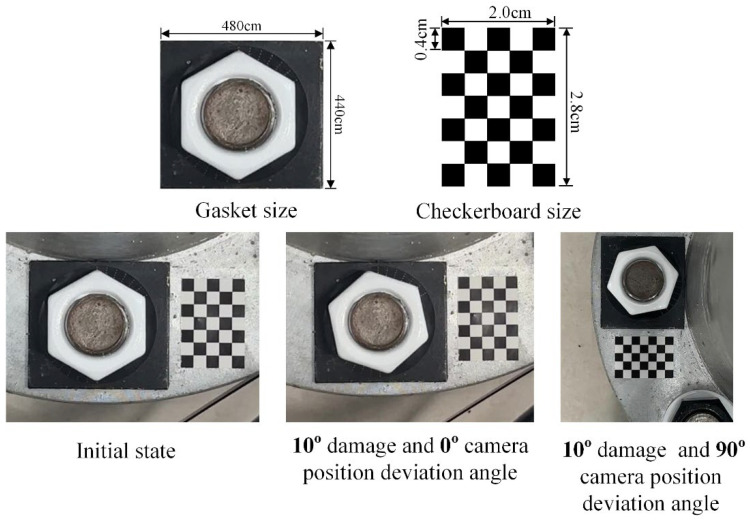
Test structure drawings and dimensions and test images.

The camera position deviation angles were set at 0° and 90°, and there was no perspective angle in the comparison test. The camera shooting height was set at 50 cm, with indoor lighting conditions. The specific experimental settings are shown in Table 2. The bolt gasket and checkerboard dimensions used in the test and the test sample drawing are shown in Figure 12.

**Figure 13:**
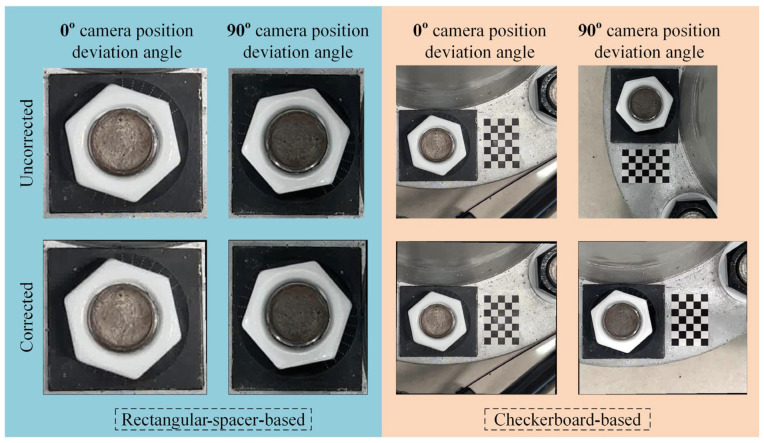
Correction results of the two correction method tests.

Using the first image under the 10° looseness condition and different camera position deviation angles, the comparison test correction results of the two methods are shown in Figure 13. As can be seen from Figure 13, the 90° camera position deviation angle could not be corrected by the existing rectangular-spacer-based correction method; however, it could be effectively corrected by the proposed checkerboard-based correction method.

**Figure 14:**
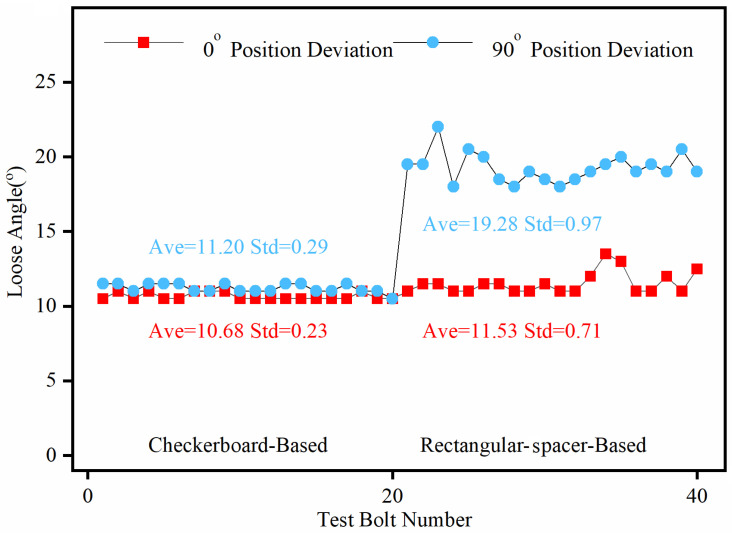
Correction results of the two correction methods.

The 10° looseness detection results of 20 images are shown in Figure 14. It can be seen from Figure 14 that when the camera position deviation is 0° after the bolt image is corrected by the two correction methods, the calculated values of the bolt-loosening angle are not much different. Still, the error of the checkerboard-based test group is lower, and the results are more stable. However, when the camera position deviates by 90°, due to the inability of the rectangular-spacer-based correction method to properly correct the bolt image, the calculated value of this set of bolt-loosening angles fluctuates greatly at around 20°, with an average value of 19.28° for the whole set, resulting in a very large error. Unlike the rectangular-spacer-based correction method, the checkerboard-based correction method can correctly rectify the bolt images, and the average value of the calculated bolt-loosening angles was only 11.2°, which is closer to the actual looseness value. Moreover, compared with the 0° camera position deviation group, the stability is not significantly reduced, and the average value is slightly increased by 0.52°.

In summary, the proposed checkerboard-based correction method exhibits obvious advantages in correction accuracy and stability compared with the existing rectangular-spacer-based correction method.

## 5. Analysis of Influencing Factors

### 5.1. Effect of Different Numbers of Correction Points

**Figure 15:**
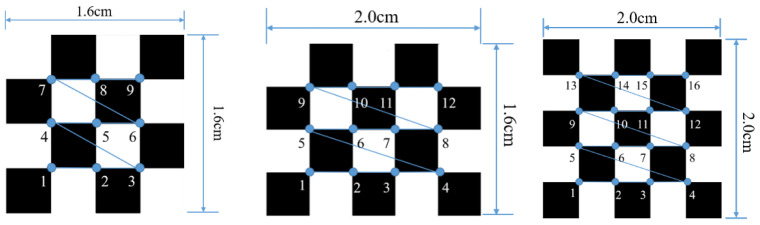
Checkerboard size chart for 9, 12, and 16 correction points.

**Figure 16:**
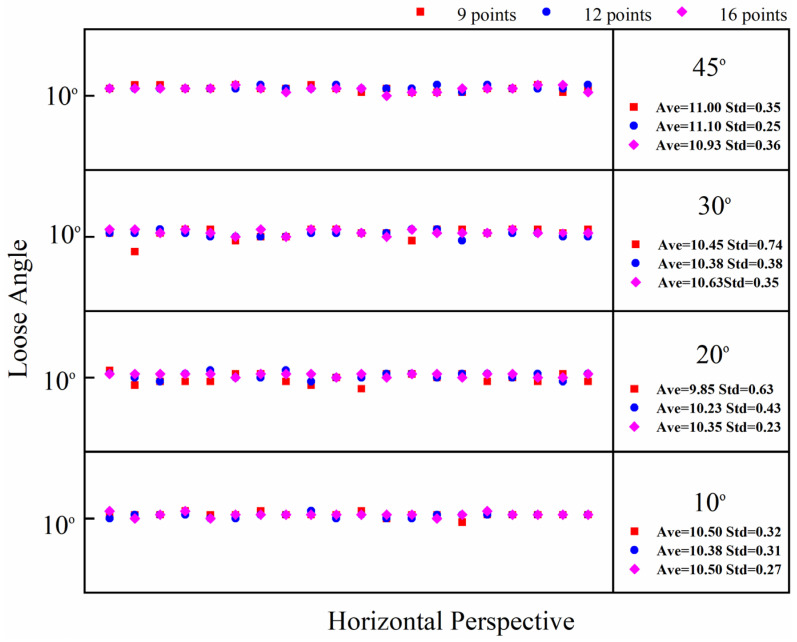
Bolt-loosening detection results of images corrected with 9, 12, and 16 correction points under horizontal perspective.

**Figure 17:**
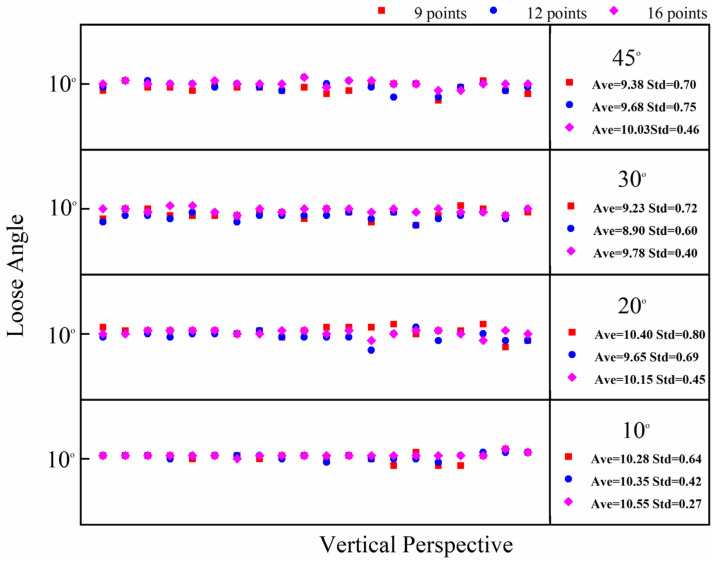
Bolt-loosening detection results of images corrected with 9, 12, and 16 correction points under vertical perspective.

**Figure 18:**
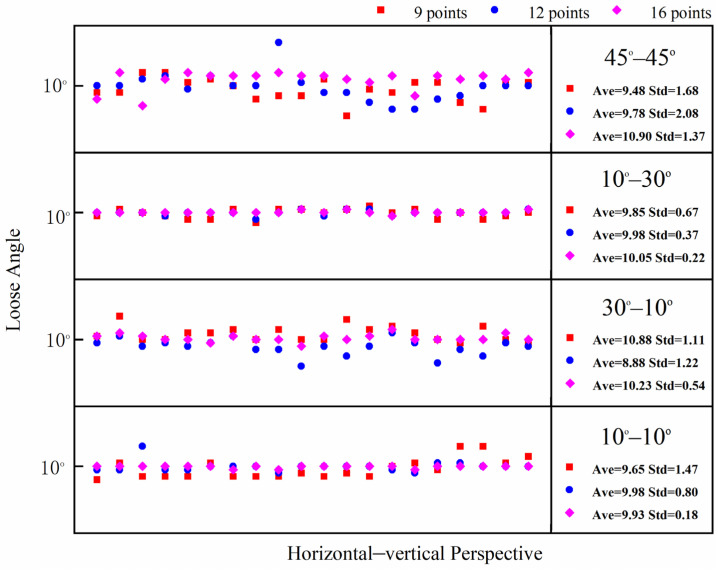
Bolt-loosening detection results of images corrected with 9, 12, and 16 correction points under horizontal–vertical perspective.

**Figure 19:**
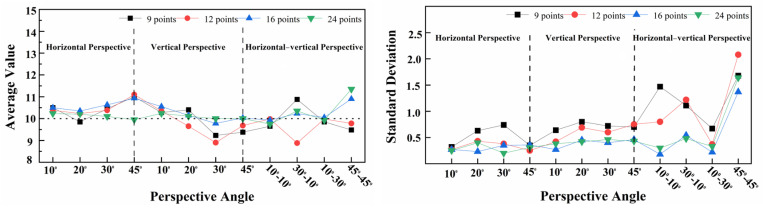
Average value and standard deviation of bolt–loosening detection results at 4 different correction points.

Flange joints have a limited bolt-to-bolt area. Therefore, when using the checkerboard to correct the bolt image, it is best to use the smallest checkerboard size to obtain accurate and stable correction results. However, different checkerboard sizes correspond to different correction points. Therefore, the influence of checkerboard correction points on the correction effect needs to be further explored, and an empirical scheme for the use of checkerboard correction points can be obtained through experiments. In comparison to a checkerboard with seven rows and five columns, this section tests the correction effect of a checkerboard with five rows and five columns, five rows and four columns, and four rows and four columns. The correction points corresponding to the three sizes of checkerboards are 16 points, 12 points, and 9 points, respectively, and the corresponding areas are 4 cm^2^, 3.2 cm^2^, and 2.56 cm^2^. The dimensions of the checkerboard are shown in Figure 15. The test conditions are set as shown in Table 3. The test results are shown in Figure 16, Figure 17 and Figure 18. The average value and standard deviation of the bolt looseness test results are shown in Figure 19.

The test results show that when the number of checkerboard correction points decreases to 16, 12, and 9, the overall average value fluctuates slightly, and the standard deviation value increases slightly. It is shown that as the number of checkerboard correction points decreases, the error fluctuation of the correction result increases gradually. When the horizontal perspective is 45°, the vertical perspective is 30°, and the horizontal–vertical perspective is 30°–10° and 45°–45°, the test results fluctuate more obviously, and the error is significantly larger than other angles. Still, the overall average error is less than 1.5°. Thus, the bolt image-correction method based on the proposed checkerboard still exhibits an excellent correction effect. It can improve the identification stability and accuracy of bolt looseness when the checkerboard contains four rows and columns and the number of correction points is nine. In actual application, a checkerboard with four rows and columns and nine correction points can be directly used.

### 5.2. The Influence of Different Camera Shooting Heights

**Figure 20:**
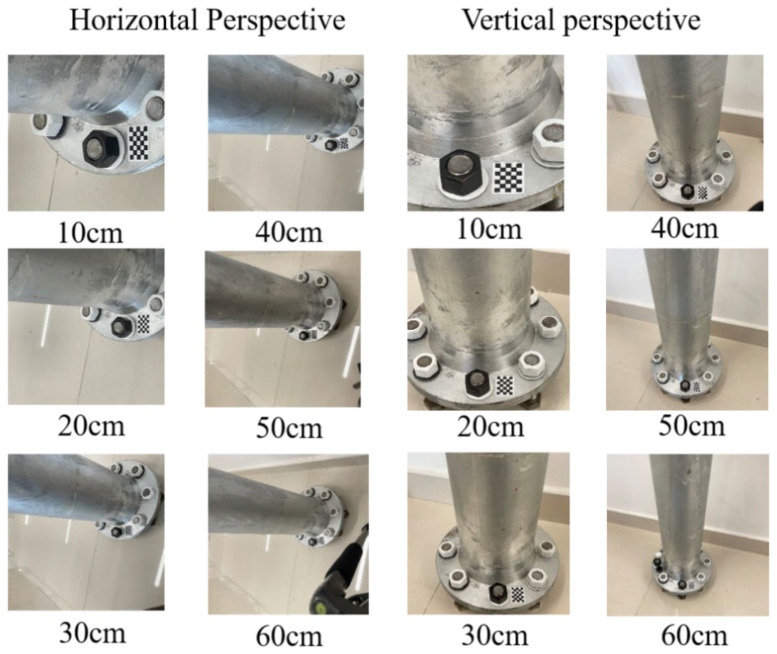
Images of bolts taken at a camera height of 10 cm to 60 cm under horizontal and vertical perspectives.

**Figure 21:**
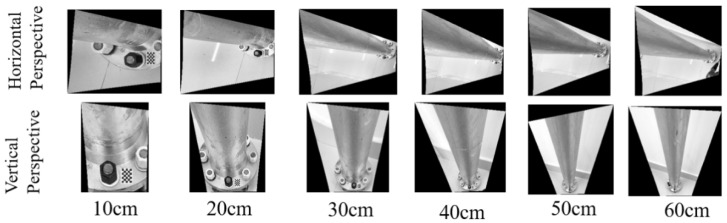
Corrected images of bolts captured from 10 cm to 60 cm shooting distances.

**Figure 22:**
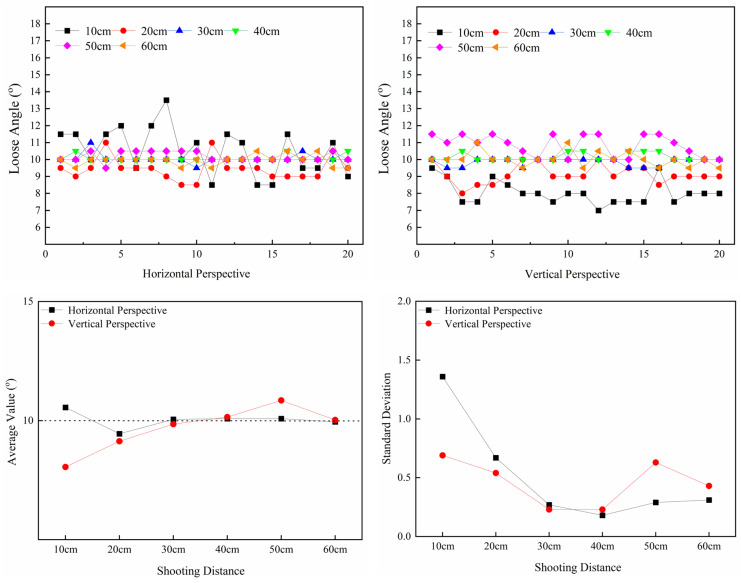
Average values and standard deviations of bolt image correction and loosening detection at camera heights from 10 cm to 60 cm.

In this section, we test the effect of bolt-loosening diagnosis after the correction of bolt images taken at different camera shooting heights. For the test setting, the bolt-loosening angle is 10^o^, and the perspective angle is 45°. We set up two groups—horizontal perspective and vertical perspective—for the experiments. The shooting heights of the camera were 10 cm, 20 cm, 30 cm, 40 cm, 50 cm, and 60 cm. Figure 20 shows the images of the bolts at camera heights ranging from 10 cm to 60 cm. The specific test conditions are shown in Table 4 below. Figure 21 is the corrected image of the bolt, which was captured from 10 cm to 60 cm shooting heights. The test results of the two groups are shown in Figure 22.

Figure 22 shows that when the camera height is less than 30 cm, the detection accuracy and stability of the bolt-loosening angle decrease. The reason is that the edge of the nut could not be clearly photographed when the distance was too close due to the influence of the high perspective angle. When the camera height ranges from 30 cm to 40 cm, the overall average is 10°, the error is the smallest, and the stability is the best. This is because a good balance is achieved between the camera height, resolution, and perspective angle. When the camera height exceeds 50 cm, the overall average value error fluctuates slightly. This is because the resolution of the cropped bolt image is low, the image-correction range is large, and the stability is poor, although the edge of the nut could be clearly photographed.

Therefore, the higher the camera shooting height, the greater the correction error of the perspective angle, resulting in the increased fluctuation of the detection results of the loosening angle. The test results show that the best distance for camera height is 30 cm to 40 cm; it is not advisable to go below 20 cm or exceed 60 cm.

### 5.3. Effect of Different Light Intensities

**Figure 23:**
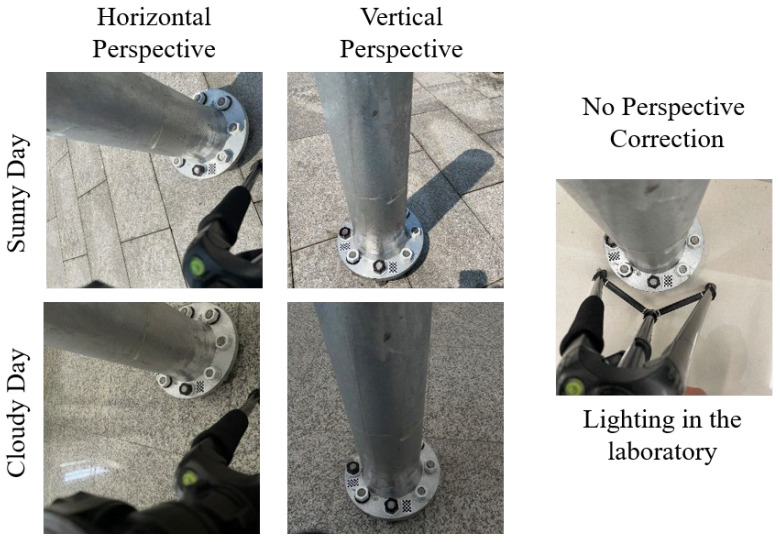
Bolt images taken under different light intensities.

**Figure 24:**
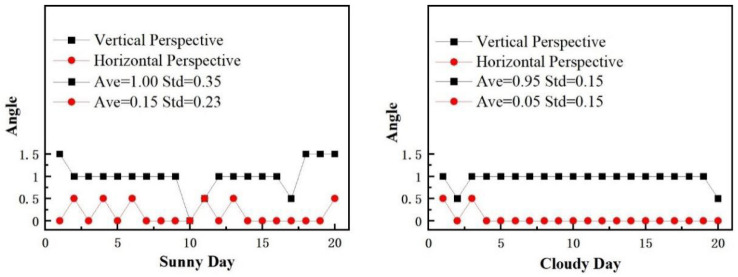
Image correction test results taken under different lighting conditions.

In order to test whether the correction method is sensitive to light intensity, images with different light intensities were tested. Two light intensity conditions, sunny and cloudy, were set up in the experiment. The test bolts were healthy bolts without loosening, and the perspective angle was 45°. The bolt image shot in the laboratory was used as the initial state, and the bolt image shot on sunny and cloudy days was used as the loose state. The loosening angle was calculated by comparing the bolt images shot on sunny and cloudy days with those shot in the laboratory. If the calculated loosening angle was equal to or close to 0°, it indicated that the light intensity exhibited no or a weak effect on the method. Detailed test parameters are presented in Table 5. Images of the bolts shot under different lighting conditions are shown in Figure 23. The test result data are illustrated in Figure 24.

Figure 24 shows that the test results on cloudy days exhibit fewer errors and better stability than those on sunny days under the same test conditions. Under the vertical perspective, the maximum difference in the bolt-loosening angle calculated by two illumination intensities is 0.5°, and the difference between the average values of the two illumination intensity tests was only 0.05°. Under the horizontal perspective, the difference between the average values of the two illumination intensity tests was 0.1°. Therefore, it can be concluded that the proposed method has low sensitivity to light intensity.

## 6. Conclusions

In this study, a bolt image-correction method for flange joints based on a checkerboard was proposed. The proposed method was verified using a prototype flange joint in the laboratory. The checkerboard is placed next to a bolt, allowing for the correction of camera position deviation and image perspective distortion based on the checkerboard’s interior corner points. The proposed method was verified using a laboratory flange prototype structure, and a comparative test was conducted with the existing rectangular gasket correction method. The test images were taken using an iPhone 12. The results show that the proposed method can effectively correct image distortion and improve the accuracy and stability of bolt-loosening detection. In order to further study the influence of the number of correction points, camera shooting distances, and light intensities, three different checkerboard specifications, six different distance values, and two light intensities were evaluated. The checkerboard specifications were five rows and five columns, five rows and four columns, and four rows and four columns. The distance values were 10 cm, 20 cm, 30 cm, 40 cm, 50 cm, and 60 cm. Sunny and cloudy days were considered. The results show that at least nine correction points are needed and that the minimum shooting distance should be between 20 cm and 60 cm. Additionally, the proposed method is weakly sensitive to light intensity. Below is a summary of the experimental results:

(1) The proposed method can correct camera position angle deviations from 0° to 180°.
(2) When the maximum perspective correction is limited to 45°, the accuracy and stability of the bolt-loosening detection results of the bolt image corrected based on the checkerboard are significantly improved compared with the uncorrected bolt image. Additionally, the average value error of the corrected bolt looseness test results is within 1.5°, and the maximum average value error of the uncorrected bolt looseness test results reaches 5.9°.
(3) The overall average value errors of the loosening diagnosis with 24, 16, 12, and 9 correction points were all less than 1.5°. This method exhibits excellent correction performance when the checkerboard has four rows, four columns, and nine correction points.
(4) When the shooting height of the camera is 30 cm to 40 cm, the overall average value error and the standard deviation are the smallest, and the angle of the bolt-loosening recognition result is more accurate and stable. When the shooting height of the camera is lower than 30 cm, the overall average value error and standard deviation gradually increase. However, when the shooting height of the camera is 10 cm, the average value error is less than 2°. When the shooting height of the camera is higher than 50 cm, although the overall average value error does not increase significantly, the standard deviation gradually increases. This indicates that data stability begins to decrease.
(5) The correction test error of the bolt image shot on a sunny day is slightly larger than that on a cloudy day. The maximum angle detected under the vertical perspective is 1.5° on a sunny day and 1° on a cloudy day. The average values of the two light intensity test results differ by 0.1° under the horizontal perspective and 0.05° under the vertical perspective. This shows that the method is less affected by light intensity and has good stability.

In summary, the proposed checkerboard-based bolt image-correction method is suitable for flange joint bolts. This method can improve the stability of flange joint bolt image correction and the accuracy and stability of bolt-loosening angle detection. Moreover, the proposed method has obvious advantages, such as excellent stability, low detection cost, and easy installation. When the proposed method is applied, it is recommended that the checkerboard be printed with waterproof and corrosion-resistant paint. Stickers are easily peeled off due to corrosion by rainwater. At the same time, detection may be affected by ice and snow.

## Figures and Tables

**Table 1:** Test conditions.

Angle of Damage (°)	Perspective Direction	Perspective Angle (°)	Number of Pictures
10	None	None	20
	Horizontal perspective	10	20
		20	20
		30	20
		45	20
	Vertical perspective	10	20
		20	20
		30	20
		45	20
	Horizontal–vertical perspective	10–10	20
		10–30	20
		30–10	20
		45–45	20

**Table 2:** Comparison test conditions.

Correction Method	Damage Angle (°)	Camera Position Deviation Angle (°)	Number of Pictures
10	0	0	1
Checkerboard-based	10	0	20
		90	20
Rectangular-spacer-based	10	0	20
		90	20

**Table 3:** Bolt-loosening detection test with 9, 12, and 16 correction points.

Angle of Damage(°)	Correction Points	Perspective Direction	Perspective Angle (°)	Number of Pictures
10	9, 12, 16	Horizontal perspective	10	20
			20	20
			30	20
			45	20
		Vertical perspective	10	20
			20	20
			30	20
			45	20
		Horizontal–vertical perspective	10–10	20
			10–30	20
			30–10	20
			45–45	20

**Table 4:** Image correction for bolt-loosening detection test at different camera heights.

Angle of Damage (°)	Perspective Direction	Perspective Angle (°)	Camera Height (cm)	Number of Pictures
10	Horizontal perspective	45	10, 20, 30, 40, 50, 60	20
	Vertical perspective	45	10, 20, 30, 40, 50, 60	20

**Table 5:** Image correction for bolt-loosening detection test under different light intensities.

Light Intensity	Perspective Direction	Perspective Angle (°)	Camera Height (cm)	Number of Pictures
Sunny day	Horizontal perspective	45	55	20
	Vertical perspective	45	55	20
Cloudy day	Horizontal perspective	45	55	20
	Vertical perspective	45	55	20

## Data Availability

All data are included in the paper.
